# SARS-CoV-2-Seronegative Subjects Target CTL Epitopes in the SARS-CoV-2 Nucleoprotein Cross-Reactive to Common Cold Coronaviruses

**DOI:** 10.3389/fimmu.2021.627568

**Published:** 2021-04-28

**Authors:** Katja G. Schmidt, Krystelle Nganou-Makamdop, Matthias Tenbusch, Boutaina El Kenz, Clara Maier, Dennis Lapuente, Klaus Überla, Bernd Spriewald, Silke Bergmann, Ellen G. Harrer, Thomas Harrer

**Affiliations:** ^1^ Infectious Disease and Immunodeficiency Section, Department of Internal Medicine 3, Universitätsklinikum Erlangen, Friedrich-Alexander-University Erlangen-Nürnberg, Erlangen, Germany; ^2^ Institute of Clinical and Molecular Virology, Universitätsklinikum Erlangen, Friedrich-Alexander University Erlangen-Nürnberg, Erlangen, Germany; ^3^ Department of Medicine 5, Universitätsklinikum Erlangen, Friedrich-Alexander-University Erlangen-Nürnberg, Erlangen, Germany

**Keywords:** SARS-CoV-2 (2019-nCoV), CD8^+^ T-cell, CTL, OC43, HKU1, CTL epitope, HIV-1

## Abstract

The beta-coronavirus SARS-CoV-2 induces severe disease (COVID-19) mainly in elderly persons with risk factors, whereas the majority of patients experience a mild course of infection. As the circulating common cold coronaviruses OC43 and HKU1 share some homologous sequences with SARS-CoV-2, beta-coronavirus cross-reactive T-cell responses could influence the susceptibility to SARS-CoV-2 infection and the course of COVID-19. To investigate the role of beta-coronavirus cross-reactive T-cells, we analyzed the T-cell response against a 15 amino acid long peptide (SCoV-DP15: DLSPRWYFYYLGTGP) from the SARS-CoV-2 nucleoprotein sequence with a high homology to the corresponding sequence (QLLPRWYFYYLGTGP) in OC43 and HKU1. SCoV-DP15-specific T-cells were detected in 4 out of 23 (17.4%) SARS-CoV-2-seronegative healthy donors. As HIV-1 infection is a potential risk factor for COVID-19, we also studied a cohort of HIV-1-infected patients on antiretroviral therapy. 44 out of these 116 HIV-1-infected patients (37.9%) showed a specific recognition of the SCoV-DP15 peptide or of shorter peptides within SCoV-DP15 by CD4^+^ T-cells and/or by CD8^+^ T-cells. We could define several new cross-reactive HLA-I-restricted epitopes in the SARS-CoV-2 nucleoprotein such as SPRWYFYYL (HLA-B*07, HLA-B*35), DLSPRWYFYY (HLA-A*02), LSPRWYFYY (HLA-A*29), WYFYYLGTGP and WYFYYLGT. Epitope specific CD8^+^ T-cell lines recognized corresponding epitopes within OC43 and HKU1 to a similar degree or even at lower peptide concentrations suggesting that they were induced by infection with OC43 or HKU1. Our results confirm that SARS-CoV-2-seronegative subjects can target SARS-CoV-2 not only by beta-coronavirus cross-reactive CD4^+^ T-cells but also by cross-reactive CD8^+^ cytotoxic T-cells (CTL). The delineation of cross-reactive T-cell epitopes contributes to an efficient epitope-specific immunomonitoring of SARS-CoV-2-specific T-cells. Further prospective studies are needed to prove a protective role of cross-reactive T-cells and their restricting HLA alleles for control of SARS-CoV-2 infection. The frequent observation of SARS-CoV-2-reactive T-cells in HIV-1-infected subjects could be a reason that treated HIV-1 infection does not seem to be a strong risk factor for the development of severe COVID-19.

## Introduction

SARS-CoV-2 is a bat-derived coronavirus which has been identified in January 2020 in patients suffering from pneumonia (1). Meanwhile, SARS-CoV-2 has emerged as a pandemic pathogen with more than 50 Million confirmed cases and more than 1.2 Million deaths worldwide (https://coronavirus.jhu.edu, assessed 09.Nov.2020). Severe coronavirus disease 2019 (COVID-19) affects mainly elderly people and patients with risk factors such as diabetes and immunosuppression, whereas the majority of patients under the age of 60 years develop only mild to moderate symptoms and up to 40% of infected subjects can even have an asymptomatic course of infection (2).

So far, the correlates of protection are not yet defined. SARS-CoV-2-specific antibodies have been detected in patients with coronavirus disease 2019 (COVID-19) usually 7 to 14 days after development of symptoms (3–5). Neutralizing antibodies against the spike protein may play an important role in the control of viral replication; however, neutralizing antibodies have not been demonstrated in all patients (4). As expected in the face of the robust antibody production, SARS-CoV-2-specific CD4^+^ T-cells have been detected in SARS-CoV-2-infected patients (4, 6–9). SARS-CoV-2-specific CD8^+^ T-cells have been reported both in patients with active COVID-19 disease (6) and in COVID-19 convalescent patients (4). Although recent reports indicated that patients with milder COVID-19 disease had stronger SARS-CoV-2-specific T-cell responses than patients with severe COVID-19 (10, 11), the contribution of SARS-CoV-2-specific CD8^+^ T-cells and their targeted epitopes for the control of SARS-CoV-2 is still undefined (12). SARS-CoV-2-specific T-cells have also been described in SARS-CoV-2-uninfected subjects (4, 6, 8, 9, 13). It has been speculated that T-cells in uninfected subjects might have been evoked by infection with circulating common cold coronaviruses (14). As the majority of published T-cell studies used mainly large SARS-CoV-2-derived peptide pools, there is still a lack of knowledge with regard to recognition of specific T-cell epitopes within the SARS-CoV-2 sequence and their potential cross-reactivity to other coronaviruses (4, 6, 9).

The nucleocapsid of SARS-CoV-2 contains a 12 amino acid long sequence (PRWYFYYLGTGP) with complete homology to the beta-coronavirus OC43 and HKU1 (**Table 1**). To investigate T-cell cross-reactivity of SARS-CoV-2 with other coronaviruses, we analyzed T-cell recognition of this homologous region using a 15 amino acid long synthetic peptide (SCoV-DP15: DLSPRWYFYYLGTGP, aa 103-107) derived from the sequence of the SARS-CoV-2 isolate Wuhan-Hu-1 (15). Analysis with the epitope prediction tool SYFPEITHI [www.syfpeithi.de (16)] revealed that SCoV-DP15 could potentially contain several T-cell epitopes. In addition to SARS-CoV-2-seronegative healthy subjects we included in our study a cohort of SARS-CoV-2-seronegative, HIV-1-infected patients, as it is still unclear whether HIV-1-infected patients on antiretroviral therapy have an increased risk of developing a severe course of COVID-19.

**Table 1:** Sequence homology within nucleocapsid proteins of SARS-CoV-2 and the human beta-coronaviruses SARS, HKU1 and OC43.

SCoV-DP15	DLSPRWYFYYLGTGP
SARS-CoV-2	MKDLSPRWYFYYLGTGPEAG
HKU1	Q-Q-L––––––––––––YAS
OC43	QRQ–L––––––––––––HAK
SARS	––E––––––––––––––––S

Shown are homologous amino acid sequences within the nucleocapsids of the coronaviruses SARS-CoV-2, HKU1, OC43 and SARS. SCoV-DP15: 15 amino acid long peptide used for peptide stimulation assays.

We could detect CD8^+^ T-cells targeting epitopes within the SCoV-DP15 peptide both in SARS-CoV-2-seronegative healthy subjects and in SARS-CoV-2-seronegative HIV-1-infected patients and we could map several CTL epitopes within this region restricted by different HLA-I-alleles. This is suggesting that cross-reactive T-cells recognizing homologous epitopes in circulating coronaviruses and in SARS-CoV-2 could play a role in the susceptibility to SARS-CoV-2 infection and COVID-19.

## Materials And Methods

### Study Subjects

Subjects for this study were recruited from healthy volunteers (n=23) and from a cohort of HIV-1-infected patients (n=116). The group of healthy blood donors consisted of 17 females and 6 males with a median age of 40 years and a range of 19 - 63 years. In the cohort of HIV-1-infected patients, the median age was 53 years with a range of 21 - 77 years. 91 of the HIV-1-infected patients were male and 25 were female. All 116 HIV-1-infected patients were on antiretroviral therapy with a median CD4 count of 692/µl (range: 21 to 1912/µl), a median CD8 count of 676/µl (range: 65 to 1742), a median CD4/CD8 ratio of 1.11 (range: 0.04 to 4.33) and a median viral load of <20 copies/ml (range: < 20 to 4100). In addition to nucleosidic and/or nucleotidic reverse transcriptase inhibitors, the antiretroviral combination regimens contained an integrase inhibitor in 88 patients, a protease inhibitor in 22 patients and a non-nucleosidic reverse transcriptase inhibitor in 20 subjects. In addition, five convalescent SARS-CoV-2-infected patients including one additional HIV-1-infected patient were investigated. These five infected patients had recovered from a mild to moderate course of COVID-19. The study with analysis of virus specific immune responses was approved from the Ethics Committee of the Medical Faculty (Number 250_15B). Blood was obtained after informed consent.

### Peptides

Peptides were synthesized as crude peptides and C-terminal acids with a purity of >70% confirmed by ESI-LCMS (EMC Microcollections, Tübingen, Germany). Peptides were dissolved in H_2_O with 10% DMSO (Merck Millipore, Darmstadt, Germany) and 1% DTT (Sigma-Aldrich). Peptide names refer to the first and last amino acid and the number of amino acids. In addition, peptides were labeled with following prefixes: SCoV: sequence derived from the SARS-CoV-2 sequence; OC43: sequence derived from the OC43/HKU1 sequence; CoV: sequence identical in SARS-CoV2, OC43 and HKU1.

### Isolation of PBMCs

Isolation of peripheral blood mononuclear cells (PBMCs) was carried out *via* density gradient centrifugation. Leucosep™ tubes (Greiner Bio One GmbH, Frickenhausen, Germany) were filled with 15ml Lymphoflot (Bio-Rad Laboratories GmbH, Feldkirchen, Germany) and centrifuged briefly to collect the fluid under the membrane. A maximum of 35ml citrate blood was transferred to the tube and filled up to 50ml with Hanks solution (Pharmacy of the University Hospital Erlangen, Erlangen, Germany), if necessary. Cells were centrifuged at 760xg for 20min and the upper layer containing PBMCs was transferred to 50ml tubes and centrifuged at 610xg for 10min. The cell pellet was then washed with 30ml Hanks solution at 610xg for 10min prior to resuspension in the appropriate cell culture medium.

### Generation of B-Lymphoblastoid Cell Lines (B-LCLs)

Following density gradient centrifugation, 10x10^6^ PBMCs were resuspended in 3-4ml of sterilely filtered supernatant from the B95-8 EBV cell line. 3µl Cyclosporine (10µg/ml) were added per 1ml and the cells were transferred to T25 flasks. After 2-3 days, R20 medium and fresh cyclosporine were added.

### Generation of SARS-CoV-2 Specific T-Cell Lines

For the generation of specific T-cell lines, 10x10^6^ PBMCs were stimulated with the peptide DLSPRWYFYYLGTGP (SCoV-DP15) or truncated peptides at a final concentration of 4 µg/ml in 1 ml R10IL2 medium consisting of RPMI 1640 medium (Sigma-Aldrich, Steinheim, Germany) with 10% heat inactivated fetal bovine serum (FCS) (PAN Biotech GmbH, Aidenbach, Germany), 1% L-glutamine (2 mmol/l), penicillin (100 U/ml), streptomycin (100 μg/ml), Hepes (10 mmol/l) (Merck KGAA, Darmstadt, Germany) and 1000 U/ml recombinant interleukin-2 (IL-2) (Proleukin, Chiron, CA, USA). After 9 to 14 days, outgrowing cells were tested for recognition of peptides by γ-Interferon Enzyme-Linked Immunospot Assays (γ-IFN ELISpot).

### Detection of SARS-CoV-2-Specific T-Cells

ELISpot assays were conducted using R5AB media consisting of RPMI 1640 medium with supplements and 5% heat-inactivated human AB-Serum (Sigma-Aldrich, Steinheim, Germany) as described (17). 96-well, nitrocellulose, filter-backed microtiter plates (MultiTrack, Lophius, Regensburg, Germany) were activated with 25 µl/well 70% methanol, and after washing twice with PBS, the plates were coated with 50 µl of anti-human γ-Interferon (γ-IFN) antibody 1-D1K (Mabtech, Stockholm, Sweden) at a final concentration of 10 µg/ml. After this, the plates were washed four times with PBS and blocked with R5AB. For the initial screening of SCov-DP15-stimulated T-cell lines, 50 µl of the cell suspension each were added to 50 µl R5AB per coated well. For further assays, cells were adjusted in R5AB medium to the desired concentration (either 1x10^5^ or 2x10^5^ cells per 100 µl) in a total volume of 100 µl per well. For analysis of freshly isolated PBMC, 2x10^5^ cells were added in 100 µl R5AB medium to the wells. Peptides were added directly to the wells at a final concentration of 20 µg/ml. Cell suspensions without peptides served as negative controls. The plates were incubated for 22 to 36 hours at 37°C and 7% CO_2_. After six washes with PBS containing 0.05% Tween-20 (PBS-T0.05%), 100 µl biotinylated anti-human γ-IFN monoclonal antibody 7-B6-1 (Mabtech, Stockholm, Sweden) was incubated at a final concentration of 2 µg/ml for two hours at room temperature. After washing six times with PBS-T0.05%, 100 µl avidin/peroxidase substrate (Vectastain^®^ Elite^®^ ABC-Kit, Linaris, Wertheim, Germany) was added to each well, the plates were incubated for one to three hours at room temperature and then washed three times with PBS-T0.05% and three times with PBS. Finally 100µl AEC substrate (3-amino-9-ethylcarbazole, Sigma-Aldrich, Germany) containing 0.06% H_2_O_2_ was added as chromogen to each well. Spots developed within ten minutes, and the reaction was stopped by washing the plates three times with distilled water. The plates were air-dried, and the spots were counted using an ELISpot reader (AID, Strassberg, Germany).

Results are reported as SFUs (Spot forming Units) either per 50µl or per cell number (1x10^5^ or 2x10^5^ cells), respectively. A peptide specific response was defined as positive if the number of spot forming units (SFUs) exceeded following thresholds: ≥ 10 SFUs and > 1.5 fold over background (SFUs without peptide). The initial screening of SCov-DP15-specific T-cells was performed in unicates, all other tests were performed in duplicates with indication of mean of duplicates.

For the assessment of the functional avidity of peptides by peptide titrations assays, peptides were added in serial dilutions ranging from 100 µg/ml to 10 ng/ml to ELISpot plates and incubated with T-cell lines (1x10^5^ per well in duplicates) for 22 – 36 h.

### HLA Restriction Analysis

HLA class I typing was performed using standard serological techniques (Biotest AG, Dreieich, Germany) or genotypic analyses (enzyme linked probe hybridization assay Biotest ELPHA, Biotest AG) according to the manufacturer’s guidelines.

HLA-I/II– restriction was demonstrated in ELISpot assays using antibodies blocking CD4, CD8 or HLA-I. The cells were incubated with a final concentration of 50 µg/ml of an anti-CD4 (OriGene Technologies, Rockville, Maryland, USA), 625 ng/ml of an anti-CD8 (BD Bioscience, San Jose, CA, USA) antibody or 20 µg/ml of an HLA-I-specific antibody (W6/32, Cymbus Biotechnology, Harrow, UK) for one hour at 37°C and 7% CO_2_. Then peptides were added directly to the wells at a final concentration of 20 µg/ml and the plates were incubated for 22 to 36 hours at 37°C in 7% CO2. In addition to ELISpot assays, CD4- or CD8-restriction was determined by intracellular cytokine staining.

The restricting HLA-I-alleles of the epitopes were determined using HLA-matched allogeneic B-LCLs or freshly isolated PBMC from HLA-I-typed blood donors. These target cells were incubated with the respective peptides for one hour and washed twice with PBS. Peptide sensitized target cells and target cells without peptides were then co-incubated with the T-cell lines in a γ-IFN ELISpot assay. If not otherwise indicated, 1x10^5^ target cells per well and 1x10^5^ T-cells were used in duplicates in these assays.

### Intracellular Cytokine Staining

4x10^5^ peptide-specific T-cell lines were stimulated with SCoV-DP15 in a final concentration of 20µg/ml for 12-14 hours. The stimulation was then carried out for an additional 4 h in the presence of 2mM Monensin (Biolegend, San Diego, CA, USA), 10ug/ml Brefeldin A (Biolegend) as well as FITC Conjugated antibodies to the granular membrane proteins CD107a (BD Biosciences, Heidelberg, Germany). Immediately following stimulation, cells were washed once and stained with LIVE/DEAD™ Fixable Aqua dye (Thermo Fisher Scientic, Waltham, MA, USA) prior to surface staining with the following anti-human monoclonal antibody reagents: anti-CD3 (AF700; BD), anti-CD4 (APC-F75; Biolegend), anti-CD8 (PerCP-Cy5.5; Biolegend) and anti-CD45RO (BV650; Biolegend). Next, cells were washed and then fixed with 2% paraformaldehyde then permeabilized with 0.5% saponin prior to staining with anti-γ-IFN (PE-Cy7; Biolegend). Finally, cells were washed and measured on an Attune NxT Flow Cytometer (Thermo Fisher Scientific) prior to data analysis using FlowJo (Becton, Dickinson and Company. Ashland OR, USA). The gating strategy is described in **Supplementary Figure S1**.

### CTL Killing Assay

To determine whether SARS-CoV-2 specific T-cell lines are able to kill target cells, a flow cytometric CTL killing assay was used. After incubation with the respective peptide, peptide-loaded PBMCs were washed three times with 15ml PBS and 0.5-1x10^6^ cells were incubated with 1 ml CFSE cell tracker (Thermo Fisher Scientific, 0.1µM in PBS) for 20min at 37°C, followed by addition of 15ml R10. After an incubation time of 5min at 37°C, cells were centrifuged at 200xg for 10min, counted and adjusted to the appropriate concentration in R10IL2. Effector and target cells were seeded at a ratio of 1:10 in 500µl R10 in a 48 well plate and incubated for 18h before staining them with propidium iodide. The percentage of CFSE- and propidium iodide positive cells representing the lysed target cells was measured using a Navios Flow Cytometer and data was analyzed using FlowJo v10.6.2 (Becton, Dickinson and Tree Star, Inc., Ashland OR, USA). The gating strategy is described in **Supplementary Figure S2**.

### Measurement of SARS-CoV-2-Antibodies

IgG- and IgM-antibodies against the SARS-CoV-2-Spike protein were first determined by a flow cytometric antibody assay measuring antibodies against human embryonic kidney cells (HEK 293T cells) transfected with either 30µg pCG1_CoV_2019-S plus 15 µg fluorescent protein (BFP) or with the control plasmid 30µg pcDNA3.1 and 15µg fluorescent protein (dsRed) as described (18). In addition, a subgroup of patients was tested with commercially available assays such as the iFlash-SARS-CoV-2 CLIA (Yhlo Biotech Co., Shenzhen, China) or the SARS-CoV-2-ELISA (Euroimmun, Lübeck, Germany).

### Quantification and Statistical Analysis

Statistical analyses were performed using the Mann–Whitney U test, the Wilcoxon Matched-Pairs Signed Ranks Test and the cross-classified table of a chi-quadrat test (Fisher exact test) using SPSS. In all analyses, p < 0.05 was considered as statistically significant.

## Results

### Recognition of the SARS-CoV-2-Derived Peptide SCoV-DP15 by SARS-CoV-2-Seronegative Subjects

**Figure 1:**
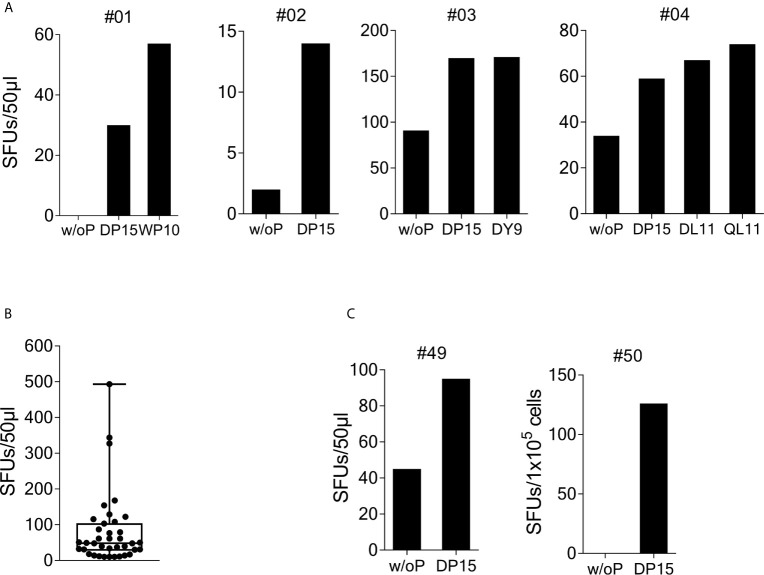
Recognition of SARS-CoV-2/OC43 homologous peptides in γ-IFN ELISpot assays. PBMCs were stimulated with SCoV-DP15 and IL-2 and after 9 to 14 days outgrowing cells were tested for recognition of SCoV-DP15 or shorter peptides within SCoV-DP15 at a final peptide concentration of 20 µg/ml. Peptide-stimulated T-cell lines without addition of peptide in the ELISpot served as negative controls to assess unspecific background reactions. Shown are SFUs per 50µl of T-cell suspensions in γ-IFN ELISpot assays. **(A)** Recognition of SCoV-DP15 or of truncated peptides by DP-15-stimulated T-cell lines from four healthy SARS-CoV-2 seronegative subjects **(B)** Magnitude of SCoV-DP15-specific response of DP-15-stimulated T-cell lines from healthy (n=3) and HIV-1-infected (n=35) CoV-DP15-responders. Shown are SFUs after subtraction of background with indication of median, range and 1^st^ and 3^rd^ quartile. **(C)** SCoV-DP15-specific γ-IFN responses of SARS-CoV-2 convalescent patient #49 and #50. 50µl of T-cells were used of donor #49, 1x10^5^ cells/well were used for donor #50. w/o. P, without peptide; SFUs, Spot Forming Units, SCoV-DP15, DLSPRWYFYYLGTGP; SCoV-DL11, DLSPRWYFYYL; OC43-QL11, QLLPRWYFYYL; CoV-WP10, WYFYYLGTGP; SCoV-DY9, DLSPRWYFY.

To investigate recognition of the SCoV-DP15 peptide, PBMCs from 23 volunteers recruited from asymptomatic hospital personnel between March and May 2020 were stimulated with peptide SCoV-DP15 and after 9 to 14 days outgrowing cells were tested for recognition of SCoV-DP15 in a γ-IFN ELISpot assay. 4 of these 23 (17.4%) subjects showed a specific recognition of SCoV-DP15 (n=3) or of the truncated peptide SCoV-DL11 within SCoV-DP15 (n=1) (**Figure 1A**). All four subjects were healthy and seronegative for SARS-CoV-2. One 59 year old subject (#1) had experienced 15 months ago a PCR-proven respiratory infection due to coronavirus OC43. Subjects #3 and #4 had an exposure to a SARS-CoV-2-infected patient without becoming infected and subject 2 had no known contacts to COVID-19 patients.

So far, it is not known yet whether HIV-1 infection and antiretroviral therapy might influence the T-cell response against SARS-CoV-2 and the beta-coronaviruses HKU-1 and OC43. Therefore, we analyzed SCoV-DP15-specific immune responses in an HLA-I-typed cohort of SARS-CoV-2-seronegative HIV-1-infected patients (samples obtained from March to June 2020) on antiretroviral therapy. The majority of these patients were recruited not randomly but patients with certain HLA-I-alleles potentially presenting predicted epitopes within SCoV-DP15 were preferentially included in the course of the study. Characteristics of SCoV-DP15-responding subjects are shown in the **Supplementary Table 1**. In peptide stimulation assays, 44 out of these 116 HIV-1-infected patients (37.9%) showed a specific response to the SCoV-DP15 peptide (n=35) or to a shorter peptide within SCoV-DP15 (n=9). There were no statistical significant differences between the HIV-1-infected 44 SCoV-DP15 responders (R) and 72 non-responders (NR) with regard to viral load (median: R: <20, NR: < 20), CD4 counts (median: R: 623/µl, NR: 734/µl), CD8 counts (median: R: 696/µl, NR: 655/µl) and CD4/CD8-ratio (median: R:1.01, NR: 1.23) (Mann-Whitney U-Wilcoxon Rank Sum Test). There was also no statistical significant difference (Fisher’s exact test) between HIV-1-infected SCoV-DP15 responder and non-responder with regard to the use of protease inhibitors (R: 23%, NR: 17%), non-nucleoside reverse transcriptase inhibitors (R: 18%, NR: 17%) or integrase inhibitors (R: 70%, NR: 79%). In total, HIV-1 infected and uninfected subjects combined, 48 of 139 subjects (34.5%) showed a response to SCoV-DP15 (n=38) or to a shorter peptide within SCoV-DP15 (n=10). There were no significant differences between the 48 responders and the 91 non-responders with regard to age (R: median: 52.5 years, NR: median: 49.5 years, Fisher´s exact test: p = not significant) and sex (R: 27% females, NR: 29.7% females, Mann-Whitney U-Wilcoxon Rank Sum Test: p = not significant). The median frequency of SCoV-DP15-reactive T-cells (n=38) in the SCoV-DP15 stimulated cells was 48.5 spot forming units (SFUs, after subtraction of the background SFUs without peptide) with a range of 10 to 493 SFUs (**Figure 1B**). All 48 responder (HIV-1-positive and HIV-1-negative subjects combined) were negative for antibodies against the SARS-CoV-2 spike protein using either a flow cytometric antibody assay measuring IgG- and IgM-antibodies (n=45), a commercially available IgG CLIA assay (iFlash-SARS-CoV-2 CLIA, Yhlo Biotech Co., Shenzhen, China, n=22) or an IgG-ELISA (Euroimmun, Lübeck, Germany, n=3).

We also analyzed five SARS-CoV-2-convalescent patients who had recovered from mild to moderate COVID-19. Two out of these five subjects (#49 and #50) responded to SCoV-DP15 (**Figure 1C**). All five convalescent patients displayed antibodies against the SARS-CoV-2 spike protein.

### CD107a Expression of SCoV-DP15-Reactive T-Cells

**Figure 2:**
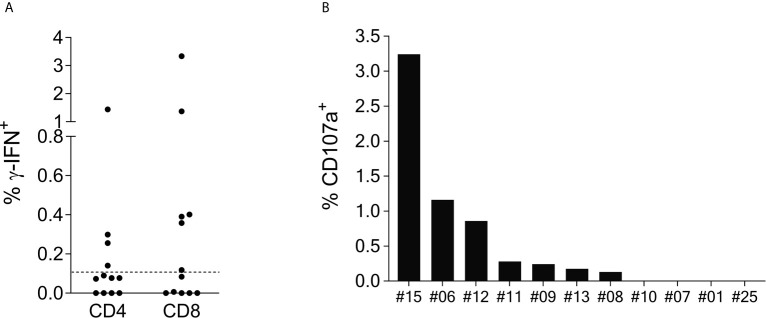
SCoV-DP15-specific T-cell lines express γ-IFN and CD107a mainly in the CD8^+^ population. **(A)**: γ-IFN expression: T-cell lines were incubated overnight with SCoV-DP15 peptide at a final concentration of 20µg/ml, followed by intracellular staining of γ-IFN (Details see methods section). T-cell lines that were incubated overnight without addition of peptides served as negative controls to assess for background γ-IFN secretion and CD107a expression. Shown are γ-IFN expressing CD4^+^ or CD8^+^ memory T-cells (CD45RO^+^). Results were considered positive when the percentage of γ-IFN positive cells after subtraction of background was >0.1% as indicated with a dashed line. **(B)** CD107a expression on CD8^+^ memory T-cells: CD107a expression was analyzed in T-cell lines in parallel to the analysis of γ-IFN secretion. CD107a responses after subtraction of background of >0.1% were considered positive.

Intracellular cytokine staining (ICS) of γ-IFN production upon SCoV-DP15 stimulation of reactive T-cell lines was performed in a subgroup of 12 SCoV-DP15-recognizing patients (11 HIV-1-infected, 1 healthy donor) 8-23 days after initial stimulation. Only in 8 out of the 12 subjects γ-IFN-secreting T-cells could be detected what can be explained by the lower sensitivity of flow cytometry in contrast to the ELISpot assay or loss of activity of cultured T-cell lines. In 6 out of the 8 responding subjects (75%) the SCoV-DP15-specific γ-IFN-secreting T-cell lines were CD8^+^ memory T-cells (**Figure 2A**). Expression of the degranulation marker CD107a on the CD8^+^ memory T-cell population could be determined in parallel in 11 of the 12 T-cell lines. After stimulation with SCoV-DP15, 7 out of 11 analyzed T-cell lines (63.6%) expressed CD107a (**Figure 2B**) indicating that these cells were indeed cytolytic effector cells (19) including two subjects without detectable γ-IFN-secretion in the ICS assay. 4 out of 12 subjects (33%) generated CD4^+^ T-cells recognizing SCoV-DP15 (**Figure 2A**). Two of these 4 subjects also displayed CD8^+^ T-cells secreting γ-IFN to SCoV-DP15 stimulation.

### Cross-Reactivity of SCoV-DL11-Recognizing T-Cell Lines to the OC43/HKU1 Peptide OC43-QL11

**Figure 3:**
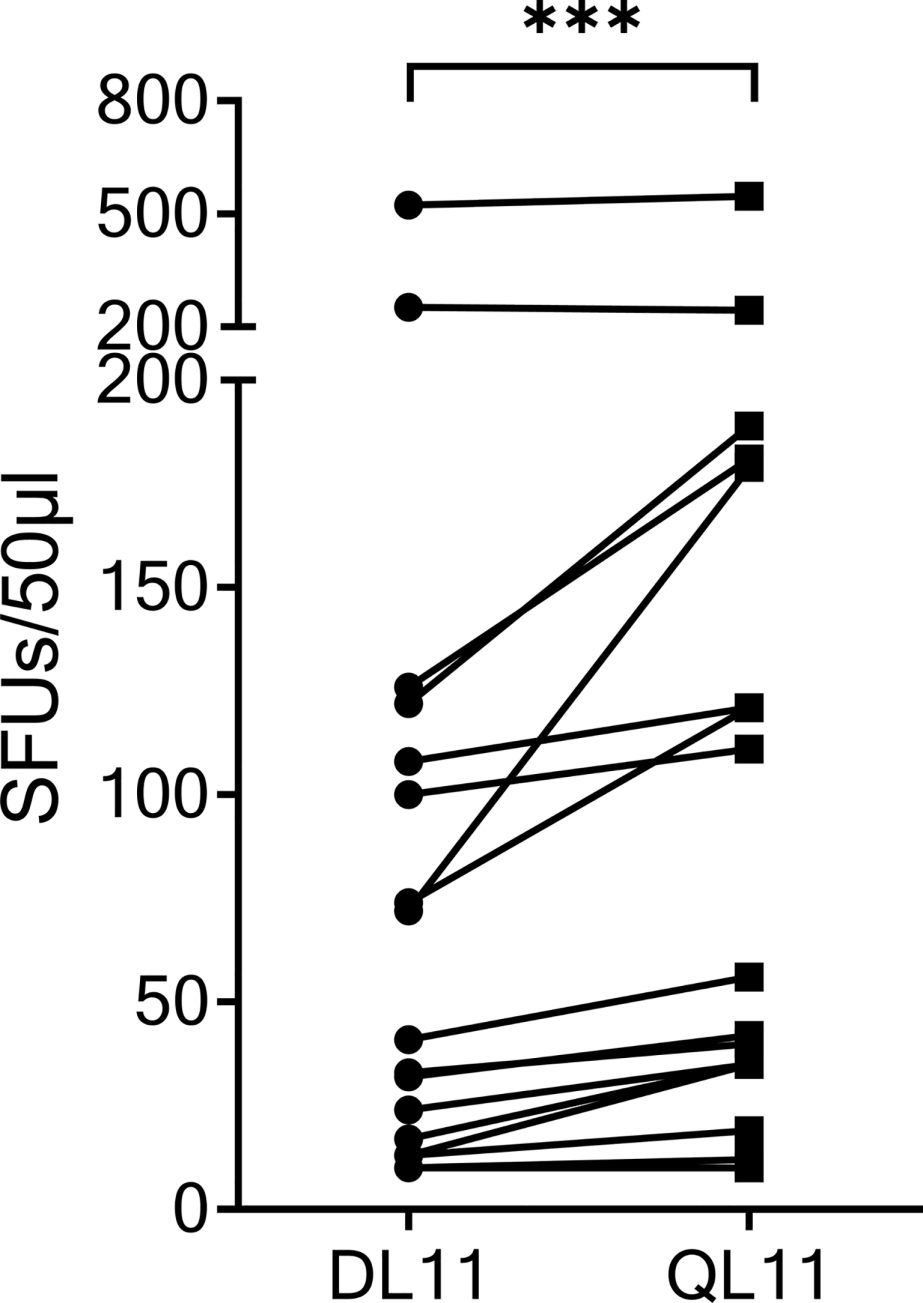
The homologous OC43 peptide OC43-QL11 was recognized better than the SARS-CoV-2 peptide SCoV-DL11. 17 T-cell lines that responded to SCoV-DL11 were tested for recognition of SCoV-DL11 and OC43-QL11 in a γ-IFN ELISpot. 50µl of each T-cell suspension were stimulated with either SCoV-DL11 or OC43-QL11 at a final peptide concentration of 20µg/ml. T-cell suspension without addition of peptides were used as negative controls to determine background responses. Shown are SFUs after subtraction of background. *** Wilcoxon Matched-Pairs Signed Ranks Test: p < 0.001.

The SCoV-DP15 sequence of the SARS-CoV-2 sequence differs from the sequence of the OC43 and HKU1 coronaviruses by a D-to-Q-substitution at position 1 and an S-to-L-substitution at position 3. To investigate the cross-reactivity of SCoV-DP15-specific T-cell lines between SARS-CoV-2 and OC43/HKU1, we analyzed 17 donor T-cell lines regarding their cross-reactivity between the truncated SARS-CoV-2-peptide SCoV-DL11 (DLSPRWYFYYL) and the homologous OC43/HKU1 peptide OC43-QL11 (QLLPRWYFYYL) in γ-IFN-ELISpot assays (**Figure 3**). Although only four of the 17 SCoV-DL11-specific T-cell lines were tested and verified to be CD8^+^ T-cells, it can be assumed that all 17 SCoV-DL11-specific T-cell lines were CD8^+^ T-cells due to the 11 amino acid length of the SCoV-DL11 peptide, as HLA-class-II binding peptides usually have an average length of 13-18 amino acids (20). The SFU frequency of OC43-QL11-responding T-cells was significantly higher than the SFU frequency of SCoV-DL11-responding T-cells (Wilcoxon Matched-Pairs Signed Ranks Test p <0.001). Out of the 17 SCoV-DL11-responding T-cell lines 17 (100%) recognized OC43-QL11 (median OC43-QL11: 56 SFUs, range 10-558, median SCoV-DL11: 41 SFUs, range 10-523) and 15 of these 17 donors displayed a higher response to the OC43/HKU1 peptide OC43-QL11 than to the SARS-CoV-2 peptide SCoV-DL11.

### Epitope Mapping of SCoV-DP15-Specific T-Cells

**Figure 4:**
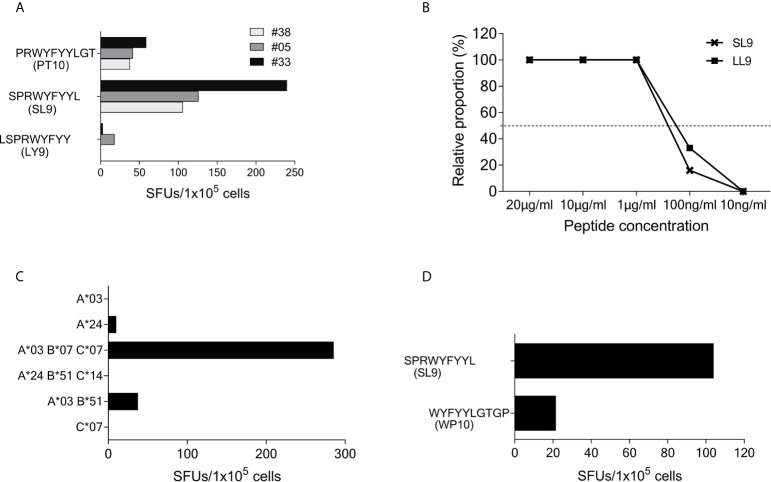
SCoV-SL9 is an HLA-B*07 and B*35 restricted epitope. **(A)** Recognition of truncated peptides overlapping with SCoV-SL9 by donors #5, #33 and #38 in a γ-IFN ELISpot assay. Shown are SFUs per 1x10^5^ cells after subtraction of background reactions in wells with T-cell suspension without peptide. **(B)** Titration of the peptides SCoV-SL9 and OC43-LL9. 1x10^5^ cells each from the T-cell line of #33 were incubated with either SCoV-SL9 or OC43-LL9 in a serial dilution with final concentrations of 20µg/ml, 10µg/ml, 1µg/ml, 100ng/ml or 10ng/ml in a γ-IFN ELISpot. SFUs are shown after subtraction of the background. Raw data are shown in **Supplementary Table S2**. **(C)** 1x10^5^ cells from the T-cell line of #5 were incubated in a γ-IFN ELISpot assay with 1x10^5^ B-LCLs loaded with the SCoV-SL9 peptide at a final concentration of 20µg/ml. Shown are SFUs after subtraction of background, which was assessed by coincubating T-cell lines with B-LCLs without addition of peptide. Shown are the HLA alleles of the B-LCLs shared by the T-cell line (HLA-I type of subject #5: A*03, A*24, B*07, B*51, C*07, C*14). **(D)** 1x10^5^ cells from the T-cell line of #41 were incubated in a γ-IFN ELISpot assay with 1x10^5^ cells of a B-LCL sharing only B*35 loaded with peptide SCoV-SL9 or the control peptide CoV-WP10 at a final concentration of 20µg/ml. Data shown represent SFUs after subtraction of background. SCoV-SL9, SPRWYFYYL; OC43-LL9, LPRWYFYYL.

**Figure 5:**
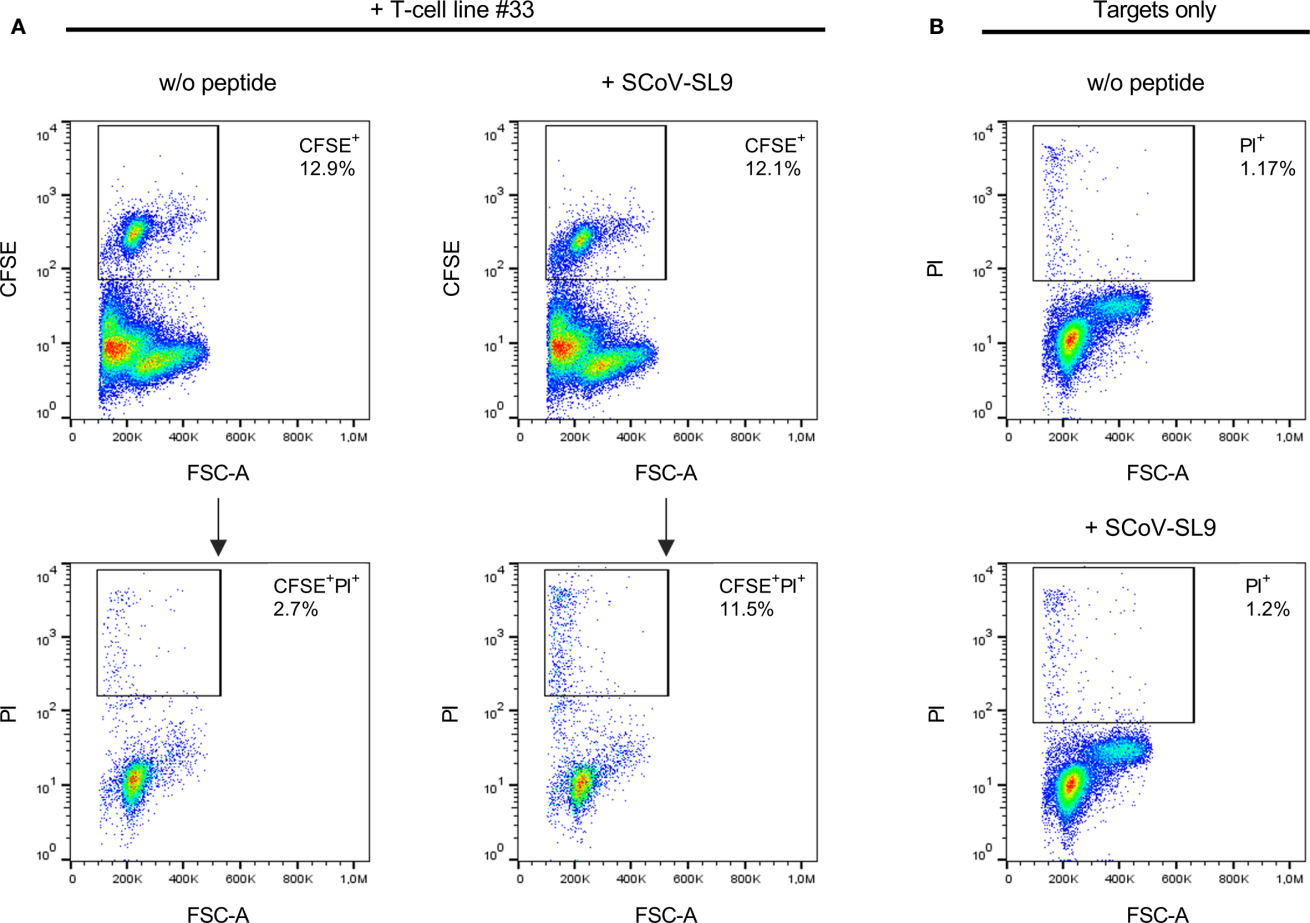
SCoV-SL9-specific T-cell lines exhibit peptide-specific cytotoxicity. **(A)** Lysis of SCoV-SL9-sensitized target cells by SCoV-SL9-specific T-cells. Autologous PBMCs from donor 33 were used as target cells and loaded with 20µg/ml SCoV-SL9 peptide (SPRWYFYYL) followed by carboxyfluoresceinsuccinimidylester. (CFSE) cell tracker staining. 3x10^4^ target cells were co-incubated with 3x10^5^ cells of a SCoV-SL9-specific T-cell line obtained from donor #33. Target cells without peptide (w/o peptide) served as control. After 18h, cells were harvested and stained with propidium iodide (PI) to assess viability and cell death (PI^+^ cells) was analyzed in the CFSE^+^ population. Shown are the percentages of CFSE^+^ target cells within the cell suspension (upper panel) as well as the percentages of PI^+^ cells in the CFSE^+^ population (lower panel: PI^+^ cells: without peptide: 2.7%, with SCoV-SL9-peptide: 11.5%). **(B)** As control for spontaneous cell death within the targets cells, 3x10^5^ target cells each were cultured either with SCoV-SL9 peptide or without peptide (w/o peptide) for 18 hours without addition of SCoV-SL9-specific T-cells. Cell death was assessed as under **(A)**. PI^+^ cells: w/o peptide: 1.17%, with SCoV-SL9 peptide: 1.20%.

Using truncated peptides we could define several HLA-I restricted T-cell epitopes: The 9-mer peptide SPRWYFYYL (SCoV-SL9) was determined to be the minimal epitope in several donors (**Figure 4A**). Blocking experiments using anti-CD8-antibodies or the anti-HLA-I-antibody w6/32 revealed that the peptide was recognized by CD8^+^ T-cells (data not shown). ELISpot assays using allogeneic EBV-transformed B-cell lines (B-LCL) or allogeneic PBMCs sharing individual HLA-I-alleles of the T-cell lines revealed that the SCoV-SL9 peptide could be presented both by HLA-B*07 (**Figure 4C**) and by HLA-B*35 (**Figure 4D**). The half-maximal peptide concentration to stimulate γ-IFN-production of SCoV-SL9-specific HLA-B*07-positive T-cell lines ranged between 1µg/ml and 100ng/ml (**Figure 4B**, **Supplementary Table S2**). SCoV-SL9-specific T-cell lines from HLA-B*07^+^ donors could lyse peptide-sensitized autologous PBMC in a flow cytometric killing assay (**Figure 5**). The SCoV-SL9 epitope is homologous in other beta-coronaviruses such as OC43 and HKU1 except for the substitution of the serine by a leucine at the P1-position. T-cell lines generated by stimulation either with SCoV-DP15 or SCoV-SL9 recognized the 9-mer peptide LPRWYFYYL (OC43-LL9) displaying a leucine at the P1 position equally well or even better than the SCoV-SL9 peptide (**Figure 4B**, **Table S2**).

**Figure 6:**
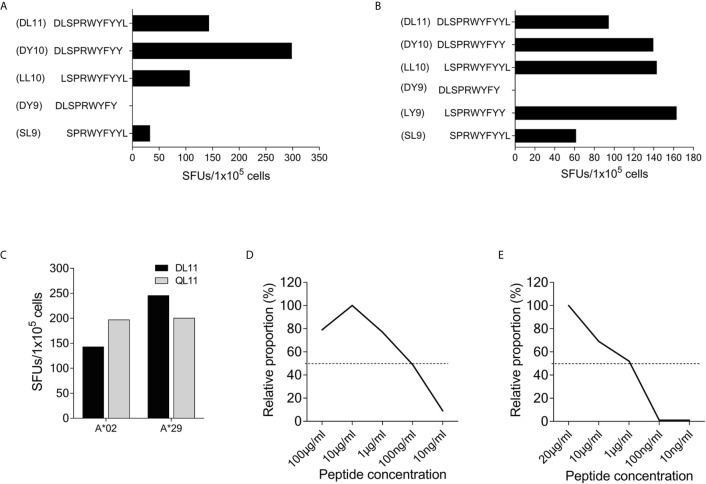
The peptide SCoV-DY10 can be presented by A*02 and A*29. **(A)** Recognition of truncated peptides by the SCoV-DP15-specific T-cell line from donor #26 in a γ-IFN ELISpot assay. 1x10^5^ T-cells were coincubated with 1x10^5^ cells of the HLA-A*02 positive T2 cell line. T2 cells without peptide served as negative controls to assess for background, SFUs are shown after subtraction of background. **(B)** Presentation of peptides by an HLA-A*29-positive B-LCL. 1x10^5^ SCoV-DP15-specific T-cells from donor #26 were co-incubated with 1x10^5^ cells of an HLA-A*29-positive B-LCL in a γ-IFN ELISpot assay. B-LCLs without addition of peptide were used as negative (=background) controls. **(C)** 1x10^5^ cells from the T-cell line #26 were incubated in a γ-IFN ELISpot assay with either the SARS-CoV-2 peptide SCoV-DL11 or the corresponding OC43 peptide OC43-QL11 at a final concentration of 20µg/ml. Shown are SFUs per 1x10^5^ cells after subtraction of background reactions in wells with T-cell suspension without peptide. **(D)** Recognition of serial dilutions of peptide SCoV-DY10 by a SCoV-DP15-stimulated T-cell line from subject #15 (HLA-A*02,A*29;B*13,B*44,C*06,C*16) in a γ-IFN ELISpot assay. Peptides were added directly to 1x10^5^ cells each of the T-cell line. The half-maximal (50%) peptide concentration is indicated by the dashed line. T2 cells without peptide served as negative controls. **(E)** Recognition of serial dilutions of peptide SCoV-DY10 presented by the A*02-positive T2 cell line (HLA-A*02,B*51,C*01). After incubation with peptides at a final concentration of 20µg/ml, T2 cells were washed thrice and added to the T-cell line from donor #26 (HLA-A*02, A*29, B*15, B*58, C*03, C*07) in a γ-IFN ELISpot assay. 1x10^5^ T2 cells and 1x10^5^ T-cells were used per well, T2 cells without peptide were used to determine background reactions. Raw data for **(D, E)** are shown in **Supplementary Table S2**.

Subject #26 (HLA-A*02, A*29, B*15, B*58, C*03, C*07) showed a strong response to SCoV-DP15 and to several truncated peptides indicating that this donor was targeting several epitopes restricted by different HLA alleles. So far, we could delineate the peptide DLSPRWYFYY (SCoV-DY10) as an epitope restricted by HLA-A*02 (**Figure 6A**) and the overlapping peptide LSPRWYFYY (SCoV-LY9) as an epitope restricted by HLA-A*29 (**Figure 6B**). Both the A*02- and the A*29-sharing cell lines presented equally well the SCoV-DY10-containing SARS-CoV-2 peptide DLSPRWYFYYL (SCoV-DL11) and the corresponding OC43-peptide QLLPRWYFYYL (OC43-QL11) demonstrating that these T-cells were cross-reactive between the SARS-CoV-2 and OC43-sequence (**Figure 6C**). The capacity of the SCoV-DY10 peptide to sensitize target cells for recognition by SCoV-DY10-specific T-cells was tested in a γ-IFN ELISpot assay applying serial dilutions of the SCoV-DY10 peptide on the HLA-A*02 and HLA-A*29-positive T-cell line of subject #15 itself. The half maximal peptide sensitizing concentration of SCoV-DY10 for recognition by SCoV-DY10-specific T-cells was determined to be approximately 100 ng/ml for the autologous T-cell line (**Figure 6D**, **Supplementary Table S2**). In addition, the peptide sensitizing capacity of SCoV-DY10 was also tested with T2-cells sharing only HLA A*02 with SCoV-DY10-specific T-cells from donor # 26. The T2-cells presented SCoV-DY10 with a half maximal peptide sensitizing concentration of 1 µg/ml (**Figure 6E**, **Supplementary Table S2**).

In subject #1 (HLA-A*01, A*11, B*08, B*35, C*04, C*07), the SCoV-DP15-stimulated T-cell line recognized the peptide WYFYYLGTGP (CoV-WP10) (**Figure 1A**). Further fine mapping of the epitope and the definition of the restricting HLA allele were not possible due to low amount of available cells.

**Figure 7:**
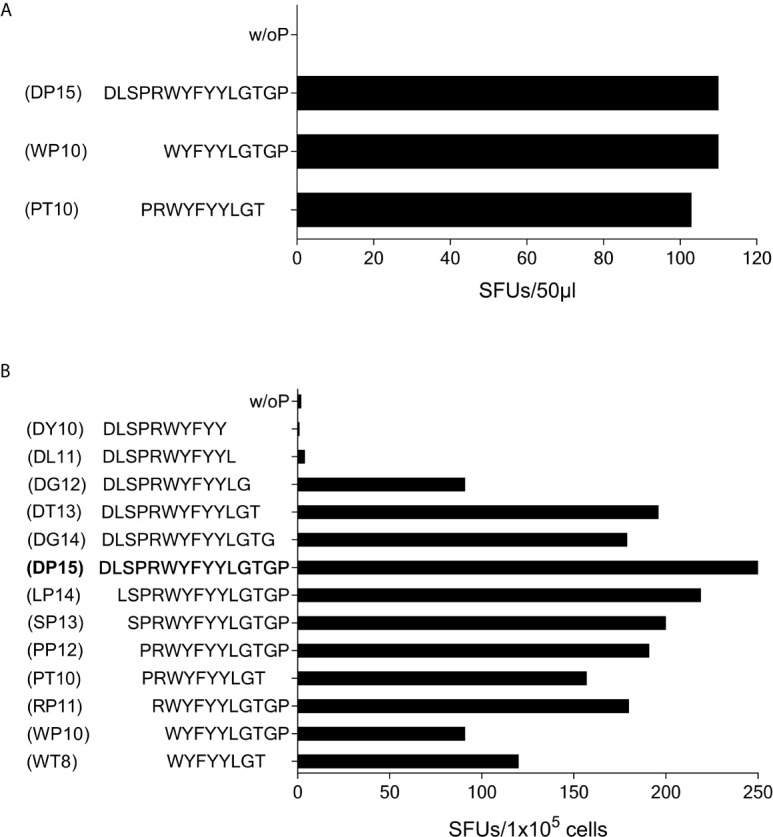
T-cell recognition of peptides CoV-PT10 and CoV-WP10. **(A)** 50µl each of the SCoV-DP15-recognizing T-cell line from Donor 7 were stimulated in a γ-IFN ELISpot assay with peptides SCoV-DP15, CoV-PT10 or CoV-WP10 at a final concentration of 20µg/ml. Shown are SFUs per well after subtraction of background values in wells without addition of peptide. **(B)** 1x10^5^ cells of the T-cell line from donor 6 were incubated in a γ-IFN ELISpot assay with peptides D15, CoV-PP12, SCoV-DG12, CoV-RP11, SCoV-DL11, CoV-WP10, CoV-PT10, CoV-WT8 at a final concentration of 20µg/ml. Responses are given as SFUs per 1x10^5^ cells after subtraction of SFUs of wells without peptide.

In subject #7 (HLA-A*01, A*03, B*13, B*35, C*04, C*18) the SCoV-DP15-stimulated cell lines recognized the 10-mer WYFYYLGTGP (CoV-WP10) and the 10-mer PRWYFYYLGT (CoV-PT10) to a similar degree (**Figure 7A**) suggesting that the 8-mer WYFYYLGT (CoV-WT8) could be the minimal epitope, however, this could not be proven yet due to a lack of sufficient T-cells for further fine mapping of the epitope and analysis of the restricting HLA allele.

Subject #6 (HLA-A*02, A*26, B*51, B*49, C*07, C*14) displayed a strong reaction against peptide SCoV-DP15 even in freshly isolated PBMC (64 SFU in 2x10^5^ PBMCs with SCoV-DP15, 1 SFU without peptide). Further epitope mapping of the SCoV-DP15-stimulated T-cell lines was performed using truncated peptides (**Figure 7B**). At the C-terminal end, omission of the proline at position 15 (peptide DG14) and of the glycine at position 14 (peptide SCoV-DT13) had no or only a slight negative effect on recognition, whereas the omission of the threonine at position 13 (peptide SCoV-DG12) reduced the number of SFUs by about one half, and further omission of the glycine at position 12 (peptide SCoV-DL11) fully abrogated peptide recognition. At the N-terminal end, omission of the first five amino acids (peptides SCoV-LP14, SCoV-SP13,CoV-PP12 CoV-PT10 and CoV-RP11) had no or only a minor effect on peptide recognition, whereas omission of the arginine at position 5 (peptide CoV-WP10) strongly diminished T-cell recognition by about one half (**Figure 7B**). Based on these results, we postulate that the SCoV-DP15-stimulated T-cell line contained two different T-cell lines recognizing two different T-cell epitopes. One minimal epitope is the 8-mer WYFYYLGT (CoV-WT8) and the second epitope contains the sequence RWYFYYLG. Further studies are necessary to define the HLA restriction of these two epitopes.

### SCoV-DP15 Recognition in Correlation to the HLA-I Type

SCoV-DP15 recognition in the SARS-CoV-2-seronegative subjects was correlated to the HLA-I type which was known in 110 of 116 (94.8%) HIV-1 patients and in 11 of 23 (47.8%) HIV-1-negative subjects. Recognition of SCoV-DP15 or one of its truncations was observed in 22 (55.3%) of 39 HLA-B*07 positive subjects, 9 (30%) of 30 of HLA-B*35 positive subjects, 16 (30.8%) of 52 HLA-A*02 positive subjects, in 3 (50%) out of 6 HLA-A*29 positive subjects. Due to biased recruitment, the frequency of HLA-B*07 and HLA-B*35 within the 110 HLA-I-typed HIV-1-infected subjects included in this study was higher (B*07: 32.7%, B*35: 25.5%) than the frequency of these HLA alleles within the 906 HIV-infected subjects of the whole Erlangen HIV cohort (B*07: 21.6%, B*35: 18%),. In contrast, the frequency of HLA-A*02 (47.2%) and HLA-A*29 (4.5%) was similar to the whole Erlangen HIV-cohort (A*02: 48.5%, A*29: 4.2%).

## Discussion

The delineation of the SARS-CoV-2-specific T-cell response is important for the understanding of the epidemiology of the SARS-CoV-2-epidemic and the development of effective SARS-CoV-2-vaccines. Using peptide SCoV-DP15 with high homology to the beta-coronaviruses OC43 and HKU1, we could detect SARS-CoV-2-reactive T-cells in a subgroup of SARS-CoV-2-seronegative asymptomatic subjects. Although SCoV-DP15 comprises only 15 amino acids from a conserved region of the nucleocapsid, 17.4% of HIV-1 negative subjects showed a positive T-cell reaction against SCoV-DP15.

So far, there are insufficient data to determine whether HIV-1-infection on antiretroviral therapy constitutes a risk factor for the acquisition of SARS-CoV-2 infection and the development of a severe course of COVID-19. Although antiviral drug therapy restores antiviral immunity in most patients in less advanced stages of HIV-1-infection, immune reconstitution may be not complete (21) and persistent T-cell dysfunction could put the patients at risk for a severe course of COVID-19. Therefore, we investigated SARS-CoV-2-reactive T-cell responses also in a cohort of HLA-I-typed HIV-1-infected subjects on antiretroviral treatment. The frequency of SCoV-DP15-recognizing subjects in the HIV-1 cohort was 37.9% and thus higher than in the HIV-1 negative subjects. However, this might be biased as patients with HLA-B*07 and HLA-B*35 were more frequently recruited after epitope mapping demonstrated associations with these HLA-I alleles. Nevertheless, we assume that this selection bias cannot fully explain the high proportion of SCoV-DP15-recognizing subjects in the HIV-1 cohort. Even on suppressive antiretroviral therapy, most of HIV-1 infected subjects show signs of T-cell activation such as elevated cytokine levels (22, 23). This could lead to a better responder rate in our peptide stimulation assay due to a lower stimulation threshold of the T-cell receptor as observed by our group using transfer of HIV-specific T-cell receptors into PBMC of HIV-1-infected patients and of healthy controls (24). In addition, PBMCs of the analyzed HIV-1 infected subjects contained in average a higher proportion of CD8^+^ T-cells as the median CD4/CD8-ratio of the HIV-1-infected subjects was 1.1 and therefore lower than the published median CD4/CD8-ratio of 1.9 in healthy subjects in Germany (25). A search for homologies between the SCoV-DP15 sequence and HIV-1 using Protein Blast [(26) https://blast.ncbi.nlm.nih.gov/Blast.cgi?PAGE=Proteins] did not reveal potentially homologous epitopes arguing against the priming of SCoV-DP15-specific T-cells by HIV-1 itself. It has been suggested that several antiretroviral drugs such as the protease inhibitor lopinavir have an antiviral effect on SARS-CoV-2 *in vitro* (27), however, clinical studies did not find a beneficial effect of lopinavir *in vivo* (28). Thus, it is highly unlikely that the HIV-1 infected subjects in our cohort had experienced asymptomatic infection with SARS-CoV-2 without developing SARS-CoV-2 antibodies due to protection by antiretroviral drugs. First reports in small cohorts suggest, that at least in treated patients in less advanced stages of HIV-1-infection, the course of COVID-19 might not be different from HIV-1 uninfected subjects (29–32). Although, it is yet unknown whether T-cell responses against the SARS-CoV-2 – derived SCoV-DP15 peptide are able to exert an antiviral effect in patients, our data indicate that beta-coronavirus – specific immune responses in HIV-1-infection are not inferior than in HIV-1 negative subjects.

Flow cytometric analyses and blocking experiments demonstrated that SCoV-DP15 was recognized both by CD4^+^ and by CD8^+^ T-cells. The expression of CD107a indicates that the CD8^+^ T-cells were indeed killer cells as it has been previously shown that the expression of CD107a on CD8^+^ T-cells is associated with their lytic function (19). Although CD107a expression was tested only in a subgroup of patients, the frequent recognition of shorter truncated peptides with a length of 11 amino acids or less underlines the high prevalence of CD8^+^ T-cells. Killing of peptide sensitized target cells by SCoV-SL9-specific CD8^+^ T-cells proved that these cells were lytic effector cells. Further experiments are necessary to investigate whether these T-cell lines are also able to lyse SARS-CoV-2 infected target cells.

Several HLA-I-restricted CTL epitopes could be mapped. The HLA-B*07/B*35-restricted epitope SPRWYFYYL (SCoV-SL9) fulfils the HLA-B*07/B*35-binding motif with a proline at the second and a leucine at the C-terminal position. The SARS-CoV-2-sequence displays a serine at the P1-position of SCoV-SL9 whereas the OC43 and HKU1 sequence shows a leucine at the P1-position. SCoV-SL9-specific T-cells recognized this epitope with a leucine at the P1 epitope equally or in some patients even better indicating that these T-cells are fully cross-reactive between SARS-CoV-2 and OC43 and HKU1. This is suggesting that SCoV-SL9-specific T-cells might have been induced by infections with OC43 and HKU1.

The 10-mer peptide DLSPRWYFYY (SCoV-DY10) was presented by HLA-A*02. For HLA-A*02, the leucine at the P2 anchor residue is a known anchor amino acid whereas the tyrosine at the C-terminal anchor position is a rarely reported anchor amino acid for HLA-A*02 [www.syfpeithi.de (16)]. This could explain the quite high 50% peptide sensitizing concentration of SCoV-DY10 indicating a low binding affinity to the HLA-A*02 molecule. The overlapping 9-mer peptide LSPRWYFYY (SCoV-LY9) was defined in our study as an HLA-A*29 restricted T-cell epitope. The C-terminal tyrosine is a known anchor for HLA-A*29.

For the other defined T-cell epitopes, the restricting HLA-allele could not yet been defined due to a limited amount of available T-cells.

All subjects with a positive response to the SARS-CoV-2-derived peptides were SARS-CoV-2-seronegative. Some had reported respiratory infections in a period of up to three months before the analysis, however, in that time period several respiratory viruses were circulating in our area including influenza A, Parainfluenza 3, rhinoviruses and coronavirus HKU1. As most SARS-CoV-2-infected subjects develop SARS-CoV-2-specific antibodies, it is highly unlikely that these respiratory infections were unrecognized SARS-CoV-2 infections.

The finding of T-cells recognizing SARS-CoV-2-sequences in seronegative subjects is suggesting that Beta-coronaviruses such as OC43 and HKU1 may induce T-cells cross-reacting to SARS-CoV-2. This is underlined by the observation that the vast majority of tested T-cell lines recognizing epitopes within the DLSPRWYFYYL sequence exhibited a stronger response to the QLLSPRWYFYYL peptide derived from the OC43/HKU1-sequence. Other epitopes such as WYFYYLGTGP, WYFYYLGT or PRWYFYYLGT displayed full sequence homology between SARS-CoV-2 and OC43 and HKU1. T-cell cross-reactivity between SARS-CoV-2 and the common cold coronaviruses OC43 and HKU1 was also reported by other groups. SARS-CoV-CD4^+^ T-cells recognizing the SARS-CoV-2-Spike protein were reported in 34% of SARS-CoV-2-seronegative healthy subjects (9). No information regarding targeted epitopes or regarding CD8^+^ T-cells were given in that report. In another study, cross-reactive CD4^+^ T-cells in unexposed seronegative subjects could be grown by peptide stimulation from purified CD4^+^ memory cells but not from naïve CD4^+^ T-cells, indicating that these cross-reactive CD4^+^ T-cells have been primed indeed by prior infections with human coronaviruses (8). In 51% of 37 SARS-CoV-2 uninfected subjects cross-reactive T-cells could be detected in peptide stimulation assays using PBMC stored prior to July 2019. This proved that SARS-CoV-2-cross-reactive T-cells could be primed by other infections (13), presumably by other human coronaviruses. In that study, one patient displayed CD4^+^ T-cells recognizing the peptide MKDLSPRWYFYYLGTGPEAG which is overlapping with the SCoV-DP15 peptide in our study. Another study reported CD4^+^ and CD8^+^ T-cell responses both in SARS-CoV-2 convalescent patients and in seronegative donors (6). Using large peptide pools and a flow cytometry based detection system, they detected SARS-CoV-2-specific CD8^+^ T-cells in 4 of 10 and SARS-CoV-2-specific CD4 cells in 6 of 10 SARS-CoV-2 unexposed donors with a positive serology for OC43. In that study, only large peptide pools had been used and no data with regard to specific epitopes have been reported. In a subsequent study, the same group reported on recognition of SARS-CoV-2-specific peptides using PBMCs collected from 2015 to 2018 demonstrating the presence of SARS-CoV-2-recognizing T-cells in the population even before the emergence of the SARS-CoV-2 epidemic (6). The presence of SARS-CoV-2-recogizing T-cells was confirmed by another group (33) describing frequent recognition of in silico defined SARS-CoV-2 epitopes by PBMCs stored prior to December 2019. This study also reported recognition of newly defined HLA-I- and HLA-II-restricted T-cell epitopes by SARS-CoV-2-infected patients and SARS-CoV-2-seronegative subjects but none of our newly defined HLA-I restricted CTL epitopes was investigated in that study. Another recent study indicated that recognition of conserved CD4 epitopes within the SARS-CoV-2 sequence correlated with a less severe course of COVID, but not the overall frequency of SARS-CoV-2 specific CD8^+^ T-cells (12). Interestingly, they found that asymptomatic SARS-CoV-2 infected patients and healthy unexposed subjects showed a different pattern of recognized HLA-I-restricted T-cell epitopes than patients with a severe COVID-19. This observation suggests that the quality of recognized CTL epitopes might be decisive for the efficacy of the SARS-CoV-2-specific CTL response. Therefore, it is an important task to delineate the various CTL epitopes for the majority of prevalent HLA-I-types in order to define the role of CTL for the control of SARS-CoV-2.

In summary, our study and the already available publications strongly suggest that SARS-CoV-2 reactive CD4^+^ and CD8^+^ T-cells are prevalent in a substantial proportion of subjects in the human populations, presumably induced by circulating common cold coronaviruses. A growing number of studies is indicating that the SARS-CoV-2-specific T-cells seem to be beneficial in patients with SARS-CoV-2 infection (4, 6–9, 12, 34). Although it is suggestive, that such pre-existing beta-coronavirus cross-reactive T-cells could influence the susceptibility to SARS-CoV-2 or the severity of COVID-19, prospective studies are needed to investigate whether the presence of cross-reactive T-cells before the acquisition of SARS-CoV-2 indeed contributes to the striking variation of the clinical course of COVID-19 in patients.

## Data Availability Statement

The raw data supporting the conclusions of this article will be made available by the authors, without undue reservation.

## Ethics Statement

The studies involving human participants were reviewed and approved by Ethics Committee of the Medical Faculty of the Friedrich-Alexander-University Erlangen-Nürnberg. The patients/participants provided their written informed consent to participate in this study.

## Author Contributions

KS and KN-M conducted the T-cell assays with support by BE and SB. CM, DL, MT, and KÜ measured the SARS-CoV-2-antibodies. BS performed HLA-I typing, EH provided patient samples and clinical data. TH designed the experiments, analyzed data, and wrote the manuscript with support of KS, KN-M, and MT. TH, KN-M, MT, und KÜ provided funding. Data of this manuscript were part of the Master´s thesis of KS. All authors contributed to the article and approved the submitted version.

## Funding

This work was supported by the German Research foundation (DFG, graduate school GRK2504/401821119, project B3: TH, KN-M, BE) and by the COVIM project of the Netzwerk Universitätsmedizin zu COVID-19: KÜ.

## Conflict of Interest

The authors declare that the research was conducted in the absence of any commercial or financial relationships that could be construed as a potential conflict of interest.
