# Supplementary material for: SARS-CoV-2-Seronegative Subjects Target CTL Epitopes in the SARS-CoV-2 Nucleoprotein Cross-Reactive to Common Cold Coronaviruses
Source: Front Immunol. 2021 Apr 28;12:627568. doi: 10.3389/fimmu.2021.627568 (PMC8113865; doi:10.3389/fimmu.2021.627568)
Supplement: Supplementary file 1 [file DataSheet_1.pdf]

Supplementary Figure 1:

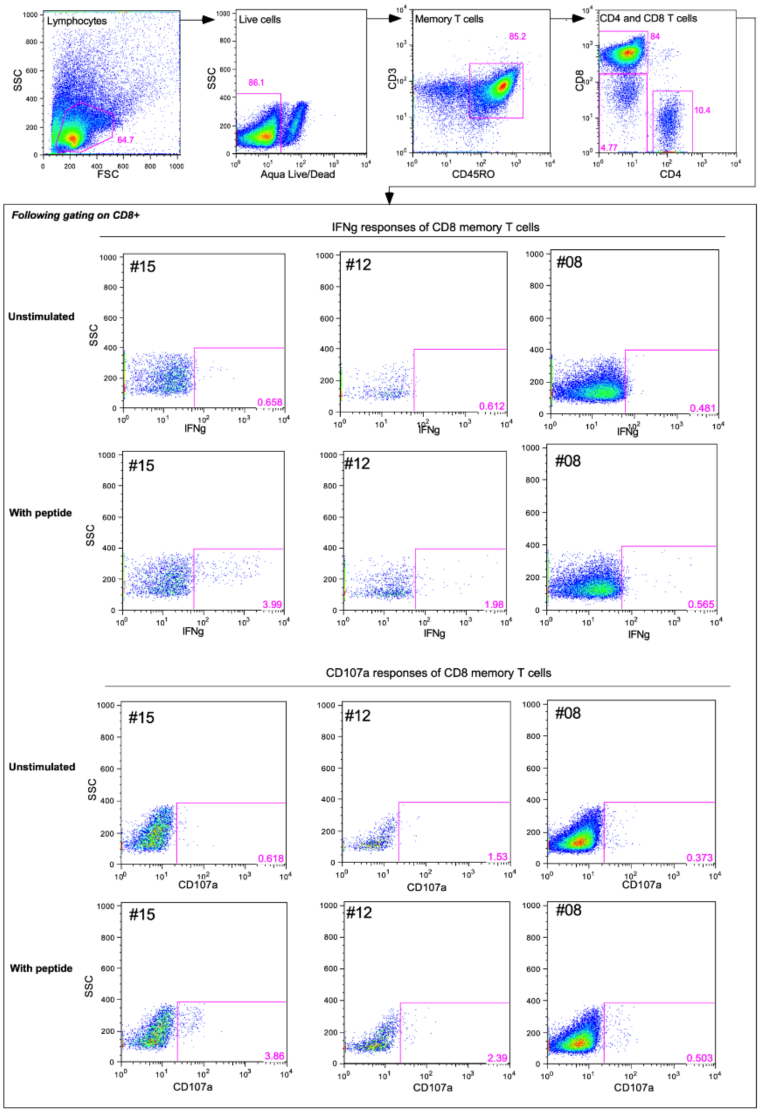

Fig. S1. Gating Strategy for intracellular cytokine staining (ICS). After gating on lymphocytes and subsequent exclusion of dead cells via viability staining memory T-cells (CD45RO$^+$) were gated. The following gate was set on CD4$^+$ or CD8$^+$ cells, in those populations the percentage of IFN-γ^+ and CD107a$^+$ cells was analyzed. Shown are representative examples of three subjects.

Supplementary Figure 2:

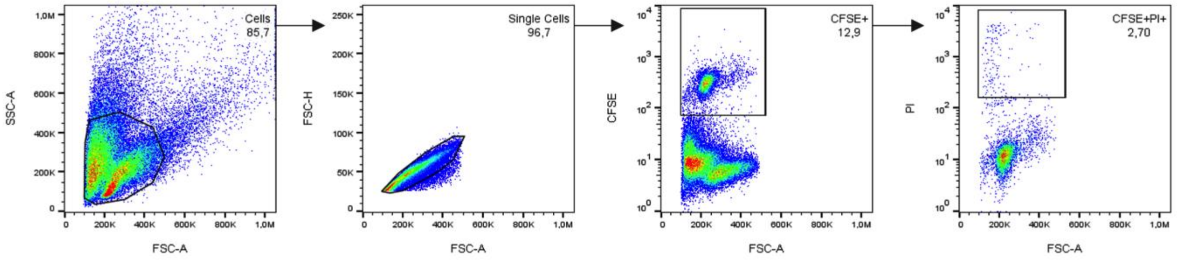

Fig. S2. Gating Strategy for CTL killing assay. After excluding debris via FSC/SSC, single cells were gated. The subsequent gate was set on CFSE^{+} cells, followed by gating of PI^{+} cells. Abbreviations: SSC: side scatter, FSC-A: forward scatter area, FSC-H: forward scatter height.

Supplementary Table 1: Characteristics of patients with recognition of SCoV-DP15.

ID	Sex	Age	Know Expo -sure	Respiratory Symptoms		HIV-Infection	Viral load	Therapy	CD4+ T-cells	CD8+ T-cells
				Current	Since 01.2020					
1	M	59	N	0	0	N	N	None	N/A	N/A
2	F	19	N	0	0	N	N	None	N/A	N/A
3	F	62	Y	0	0	N	N	None	N/A	N/A
4	F	39	Y	0	0	N	N	None	N/A	N/A
5	M	24	N	0	0	Y	<20	LPV/r/RAL/RPV	1282	709
6	F	57	N	0	0	Y	<20	FTC/TAF/COB/ EVG	556	519
7	M	58	N	0	0	Y	<20	FTC/TAF/COB/ EVG	554	316
8	M	37	N	PA	0	Y	<20	DRV/COB/FTC/ TAF, RPV	520	1301
9	M	54	N	0	0	Y	20	FTC/TDF, EFV	999	529
10	F	29	N	0	0	Y	<20	FTC/TDF, RAL	701	684
11	M	54	N	0	0	Y	<20	FTC/TAF/COB/ EVG	797	708
12	M	38	N	0	0	Y	<20	FTC/TAF/COB/ EVG	795	867
13	F	58	N	0	0	Y	50	BIC/FTC/TAF	1114	836
14	M	68	N	0	0	Y	<20	BIC/FTC/TAF	728	507
15	M	66	N	0	0	Y	<20	BIC/FTC/TAF	348	218
16	F	49	N	0	CC, ST	Y	<20	EFV/FTC/TDF	1075	358
17	M	40	N	0	0	Y	<20	BIC/FTC/TAF	781	1011
18	M	44	N	0	0	Y	20	DRV/r, DTG,ETR	630	1013
19	F	45	N	0	0	Y	20	BIC/FTC/TAF	496	848
20	M	63	N	0	0	Y	<20	ABC/3TC/DTG	309	452
21	F	37	N	0	FI 01/20	Y	<20	DRV/COB/FTC/ TAF	484	405
22	F	58	N	0	0	Y	<20	FTC/TDF,RAL	390	205
23	F	39	N	C, R, SB	C, R, SB	Y	<20	BIC/FTC/TAF	232	392
24	F	39	N	0	0	Y	<20	DRV/r, FTC/TDF	487	578
25	M	59	N	0	0	Y	60	FTC/TAF/COB/ EVG	370	773
26	M	49	N	DC	FI 02/20	Y	20	BIC/FTC/TAF	650	729

27	F	30	N	0	C, F 02/20	Y	<20	DRV/r, FTC/TDF	907	774
28	M	36	N	0	0	Y	<20	BIC/FTC/TAF	540	526
29	M	63	N	0	0	Y	<20	BIC/FTC/TAF	381	1397
30	M	55	N	C, Ho	FL 03/20	Y	<20	BIC/FTC/TAF	996	1127
31	M	63	N	0	0	Y	20	ABC/3TC/DTG	311	346
32	M	66	N	0	0	Y	<20	FTC/TAF/BIC	584	584
33	M	49	N	0	RI 03/20	Y	<20	ABC/3TC/DTG	987	751
34	M	52	N	0	0	Y	<20	FTC/TAF/BIC	950	1130
35	M	45	N	0	0	Y	<20	FTC/TAF/COB/ EVG	516	937
36	M	38	N	0	0	Y	<20	DRV/COB/FTC/ TAF	691	549
37	M	54	N	0	0	Y	<20	ABC/3TC,EFV	765	659
38	F	57	N	0	RI 03/20	Y	<20	ABC/3TC,RAL	382	973
39	M	59	N	RA	CC, ST 03/20	Y	<20	FTC/TAF,AZV/r DTG	696	480
40	M	54	N	RA	0	Y	<20	FTC/TAF/BIC	421	723
41	M	31	N	0	0	Y	<20	FTC/TAF/BIC	1166	768
42	M	43	N	0	0	Y	<20	FTC/TDF/EFV	615	476
43	M	52	N	0	0	Y	<20	ABC/3TC, EFV	634	888
44	M	70	N	0	0	Y	20	DRV/r,FTC/TAF, DTG	183	711
45	M	53	N	0	0	Y	<20	FTC/TAF/BIC	417	544
46	M	56	N	0	0	Y	<20	DRV/r,FTC/TAF	329	200
47	M	52	N	0	0	Y	20	ABC/3TC/DTG	972	1372
48	M	56	N	0	0	Y	<20	FTC/TDF,RPV	658	493
49	F	62	I	**COVID-19 04/20**	0	N	N	None	540	132
50	M	56	I	D	**COVID-19 04/20**	Y	<20	FTC/TAF/BIC	1034	643

Abbreviations: M: male, F: female; I: infected: patients #49 and #50 had COVID-19. N: No, Y: Yes, AL: aching limbs, C: cough, CC: common cold, D: slight cyspnoe, DC: dry cough, F: fever, FI: flu-like infection, H: headache, Ho: hoarseness, PA: pollen allergy, R: rhinitis, RA: rhinitis allergica, RI: respiratory infection, SB: shortness of breath, ST: sore throat.

Supplementary Table 2: Raw data of peptide titration experiments.

	100µg/ml	20µg/ml	10µg/ml	1µg/ml	100ng/ml	10ng/ml	w/o. peptide
**SL9**		2000	2000	2000	390	64.5	83
**LL9**		2000	2000	2000	726.5	82.5	86.5
**DY10**	244.5		283.5	240.5	190	115.5	98.5
**T2 + DY10**		159	109	82	1	1	1

Raw data of peptide titration experiments depicted in figures 4B, 6D and 6E. Shown are means of duplicates of SFUs at the indicated peptide concentrations per $1x10^5$ cells in the ELISpot assays. DY10 (SCoV-DY10) titration of T-cells from subject #15 corresponds to figure 6D. Titration data of T cell line #26 with DY10 presented by the T2 cell line corresponds to figure 6E. SL9 (SCoV-SL9) and LL9 (OC43-LL9) titration data of T cell line from donor #33 correspond to the graph in figure 4B.
