# Supplementary material for: SARS-CoV-2-Seronegative Subjects Target CTL Epitopes in the SARS-CoV-2 Nucleoprotein Cross-Reactive to Common Cold Coronaviruses
Source: Front Immunol. 2021 Apr 28;12:627568. doi: 10.3389/fimmu.2021.627568 (PMC8113865; doi:10.3389/fimmu.2021.627568)
Supplement: Supplementary file 2 [file Table_1.docx]

**Supplementary Table 1: Characteristics of patients with recognition of SCoV-DP15.**

| **ID** | **Sex** | **Age** | **Know Expo-sure** | **Respiratory Symptoms** | | **HIV-Infection** | **Viral load** | **Therapy** | **CD4+ T-cells** | **CD8+ T-cells** |
| --- | --- | --- | --- | --- | --- | --- | --- | --- | --- | --- |
|  |  |  |  | Current | Since 01.2020 |  |  |  |  |  |
| 1 | M | 59 | N | 0 | 0 | N | N | None | N/A | N/A |
| 2 | F | 19 | N | 0 | 0 | N | N | None | N/A | N/A |
| 3 | F | 62 | Y | 0 | 0 | N | N | None | N/A | N/A |
| 4 | F | 39 | Y | 0 | 0 | N | N | None | N/A | N/A |
| 5 | M | 24 | N | 0 | 0 | Y | <20 | LPV/r/RAL/RPV | 1282 | 709 |
| 6 | F | 57 | N | 0 | 0 | Y | <20 | FTC/TAF/COB/  EVG | 556 | 519 |
| 7 | M | 58 | N | 0 | 0 | Y | <20 | FTC/TAF/COB/  EVG | 554 | 316 |
| 8 | M | 37 | N | PA | 0 | Y | <20 | DRV/COB/FTC/  TAF, RPV | 520 | 1301 |
| 9 | M | 54 | N | 0 | 0 | Y | 20 | FTC/TDF, EFV | 999 | 529 |
| 10 | F | 29 | N | 0 | 0 | Y | <20 | FTC/TDF, RAL | 701 | 684 |
| 11 | M | 54 | N | 0 | 0 | Y | <20 | FTC/TAF/COB/  EVG | 797 | 708 |
| 12 | M | 38 | N | 0 | 0 | Y | <20 | FTC/TAF/COB/  EVG | 795 | 867 |
| 13 | F | 58 | N | 0 | 0 | Y | 50 | BIC/FTC/TAF | 1114 | 836 |
| 14 | M | 68 | N | 0 | 0 | Y | <20 | BIC/FTC/TAF | 728 | 507 |
| 15 | M | 66 | N | 0 | 0 | Y | <20 | BIC/FTC/TAF | 348 | 218 |
| 16 | F | 49 | N | 0 | CC, ST | Y | <20 | EFV/FTC/TDF | 1075 | 358 |
| 17 | M | 40 | N | 0 | 0 | Y | <20 | BIC/FTC/TAF | 781 | 1011 |
| 18 | M | 44 | N | 0 | 0 | Y | 20 | DRV/r, DTG,ETR | 630 | 1013 |
| 19 | F | 45 | N | 0 | 0 | Y | 20 | BIC/FTC/TAF | 496 | 848 |
| 20 | M | 63 | N | 0 | 0 | Y | <20 | ABC/3TC/DTG | 309 | 452 |
| 21 | F | 37 | N | 0 | FI 01/20 | Y | <20 | DRV/COB/FTC/  TAF | 484 | 405 |
| 22 | F | 58 | N | 0 | 0 | Y | <20 | FTC/TDF,RAL | 390 | 205 |
| 23 | F | 39 | N | C, R, SB | C, R, SB | Y | <20 | BIC/FTC/TAF | 232 | 392 |
| 24 | F | 39 | N | 0 | 0 | Y | <20 | DRV/r, FTC/TDF | 487 | 578 |
| 25 | M | 59 | N | 0 | 0 | Y | 60 | FTC/TAF/COB/  EVG | 370 | 773 |
| 26 | M | 49 | N | DC | FI 02/20 | Y | 20 | BIC/FTC/TAF | 650 | 729 |
| 27 | F | 30 | N | 0 | C, F 02/20 | Y | <20 | DRV/r, FTC/TDF | 907 | 774 |
| 28 | M | 36 | N | 0 | 0 | Y | <20 | BIC/FTC/TAF | 540 | 526 |
| 29 | M | 63 | N | 0 | 0 | Y | <20 | BIC/FTC/TAF | 381 | 1397 |
| 30 | M | 55 | N | C, Ho | FL 03/20 | Y | <20 | BIC/FTC/TAF | 996 | 1127 |
| 31 | M | 63 | N | 0 | 0 | Y | 20 | ABC/3TC/DTG | 311 | 346 |
| 32 | M | 66 | N | 0 | 0 | Y | <20 | FTC/TAF/BIC | 584 | 584 |
| 33 | M | 49 | N | 0 | RI 03/20 | Y | <20 | ABC/3TC/DTG | 987 | 751 |
| 34 | M | 52 | N | 0 | 0 | Y | <20 | FTC/TAF/BIC | 950 | 1130 |
| 35 | M | 45 | N | 0 | 0 | Y | <20 | FTC/TAF/COB/  EVG | 516 | 937 |
| 36 | M | 38 | N | 0 | 0 | Y | <20 | DRV/COB/FTC/  TAF | 691 | 549 |
| 37 | M | 54 | N | 0 | 0 | Y | <20 | ABC/3TC,EFV | 765 | 659 |
| 38 | F | 57 | N | 0 | RI 03/20 | Y | <20 | ABC/3TC,RAL | 382 | 973 |
| 39 | M | 59 | N | RA | CC, ST 03/20 | Y | <20 | FTC/TAF,AZV/r DTG | 696 | 480 |
| 40 | M | 54 | N | RA | 0 | Y | <20 | FTC/TAF/BIC | 421 | 723 |
| 41 | M | 31 | N | 0 | 0 | Y | <20 | FTC/TAF/BIC | 1166 | 768 |
| 42 | M | 43 | N | 0 | 0 | Y | <20 | FTC/TDF/EFV | 615 | 476 |
| 43 | M | 52 | N | 0 | 0 | Y | <20 | ABC/3TC, EFV | 634 | 888 |
| 44 | M | 70 | N | 0 | 0 | Y | 20 | DRV/r,FTC/TAF,DTG | 183 | 711 |
| 45 | M | 53 | N | 0 | 0 | Y | <20 | FTC/TAF/BIC | 417 | 544 |
| 46 | M | 56 | N | 0 | 0 | Y | <20 | DRV/r,FTC/TAF | 329 | 200 |
| 47 | M | 52 | N | 0 | 0 | Y | 20 | ABC/3TC/DTG | 972 | 1372 |
| 48 | M | 56 | N | 0 | 0 | Y | <20 | FTC/TDF,RPV | 658 | 493 |
| 49 | F | 62 | I | **COVID-19 04/20** | 0 | N | N | None | 540 | 132 |
| 50 | M | 56 | I | D | **COVID-19 04/20** | Y | <20 | FTC/TAF/BIC | 1034 | 643 |

Abbreviations: M: male, F: female; I: infected: patients #49 and #50 had COVID-19. N: No, Y: Yes, AL: aching limbs, C: cough, CC: common cold, D: slight cyspnoe, DC: dry cough, F: fever, FI: flu-like infection, H: headache, Ho: hoarseness, PA: pollen allergy, R: rhinitis, RA: rhinitis allergica, RI: respiratory infection, SB: shortness of breath, ST: sore throat.
